# Novel in-frame deletion in *MFSD8* gene revealed by trio whole exome sequencing in an Iranian affected with neuronal ceroid lipofuscinosis type 7: a case report

**DOI:** 10.1186/s13256-018-1788-7

**Published:** 2018-09-25

**Authors:** Ali Hosseini Bereshneh, Masoud Garshasbi

**Affiliations:** Department of Medical Genetics, Faculty of Medical Sciences, Tarbiat Modares University, Tehran, Iran

**Keywords:** Neuronal ceroid lipofuscinosis type 7, N.eurodegenerative, *MFSD8* gene

## Abstract

**Background:**

The neuronal ceroid lipofuscinoses are a group of neurodegenerative, lysosomal storage disorders. They are inherited as an autosomal recessive pattern with the exception of adult neuronal ceroid lipofuscinosis, which can be inherited in either an autosomal recessive or an autosomal dominant manner. The neuronal ceroid lipofuscinoses are characterized by accumulation of autofluorescent lipopigments in the cells and one of the most important pathological manifestations is ceroid accumulation in the lysosomes. Various types of neuronal ceroid lipofuscinoses are categorized based on the clinical manifestations and the genes involved. Accumulatively, 15 different genes have been found so far to be implicated in the pathogenesis of at least nine different types of neuronal ceroid lipofuscinoses, which result in similar pathological and clinical manifestations.

**Case presentation:**

A 5-year-old Iranian boy affected by a neurodegenerative disorder with speech problems, lack of concentration, walking disability at age of 4 years leading to quadriplegia, spontaneous laughing, hidden seizure, clumsiness, psychomotor delay, and vision deterioration at age of 5 years, which could be the consequence of macular dystrophy, was referred to us for genetic testing. Trio whole exome sequencing, Sanger validation, and segregation analysis discovered a novel in-frame small deletion c.325_339del (p.Val109_Ile113del) in *MFSD8* gene associated with neuronal ceroid lipofuscinosis type 7.

**Conclusions:**

The deletion found in this patient affects the exon 5 of this gene which is the region encoding transmembrane domain. Sequencing analysis in this family has shown that the index is homozygous for 15 base pairs in-frame deletion, his uncle has normal homozygous, and his parents are heterozygous. This pattern of mutation inheritance and the signs and symptoms observed in the affected male of this family are compatible with what is described in the literature for neuronal ceroid lipofuscinosis type 7 and, therefore, suggest that the *MFSD8* gene deletion found in this study is most probably the cause of disease in this family.

## Background

The neuronal ceroid lipofuscinoses (NCLs or CLNs), also called Batten disease, are a set of neurodegenerative genetic disorders with an overall frequency of 1 in 100,000 individuals around the world [1, 2]. This condition conforms to an autosomal recessive (AR) pattern of inheritance and is mainly caused by consanguineous marriage [3]. Babies affected with these lysosomal storage diseases do not manifest any clinical symptoms at birth; however, signs and symptoms mostly begin before 7 years of age depending on the gene involved [3, 4]. These conditions are genetically heterogeneous and consist of a wide spectrum of phenotypical and clinical manifestations. NCLs are characterized by the accumulation of autofluorescent lipopigments in cells [5] and one of the most important pathological manifestations is ceroid accumulation in lysosomes [1, 6]. The accumulation of storage material in nerve cells results in massive neuronal loss and subsequent apoptosis, as has been shown in an autopsy of the brain [7]. The categorization of various types of NCLs is based on the phenotype and the genes that are mutated. Accumulatively, mutation in at least 15 different genes has been shown to be implicated in the pathogenesis of at least nine different types of NCLs which result in a similar pathological and clinical manifestation; Table 1 [8–10]. Genes involved in the pathogenesis of NCLs are mainly affected by loss-of-function mutations [11, 12].

**Table 1:** Different types of neuronal ceroid lipofuscinosis

Types of NCLs	Pattern	Onset	Gene	Chromosome location
NCL1	AR	Infantile Adulthood (mild form)	*PTT1*/*CLN1*	1p32
NCL2	AR	Late infantile	*TPP1*/*CLN2*	11p15.5
NCL3		Juvenile	*CLN3*	16p12.1
NCL4	AR AD	Adulthood	*CLN6* *DNAJC5*	15q21-q23 20q13.33
NCL5	AR	Late infantile Adulthood (mild form)	*CLN5*	13q21.1-q32
NCL6	AR	Late infantile	*CLN6*	15q21.q23
NCL7	AR	Late infantile	*CLN7*/*MFSD8*	4q28.1-q28.2
NCL8	AR	Late infantile	*CLN8*	8p23.3
NCL9	AR	Juvenile	Unknown	Unknown
NCL10	AR	Newborn Adulthood (mild form)	*CLN10*/*CTSD*	1p15.5

*AD* autosomal dominant, *AR* autosomal recessive, *NCL* neuronal ceroid lipofuscinosis

Phenotypically, there are three common different forms of NCLs; the categorization of these three forms of NCLs is mainly based on the age of onset: infantile NCL (INCL) that begins between 6 and 18 months of age; late INCL (LINCL) which begins at 2–4 years of age; and juvenile NCL (JNCL) which begins at the age of 6–10 years or sometimes in adulthood [13–15]. All types show overlapping sign and symptoms, mainly cognitive impairment, progressive neuronal degeneration, motor deficits, seizures, progressive visual deterioration, and blindness [16]. Motor dysfunction could lead to subsequent quadriplegia [17, 18]. The genes involved in NCLs present similar clinical manifestations; so, the finding of genetic mutations may explain unclear clinical findings and distinguish between them [19, 20].

The most prevalent types of NCLs are NCL1, which is considered an infantile form, NCL2, which is considered a late infantile form, and NCL3, which is considered a juvenile form [21]. NCL1 is the most common form of INCLs. At birth, a baby with NCL1 has normal development, but this process is lost and motor dysfunction leads to hypotonia [22]. There is evident vision loss and seizure at approximately 1 year of age [23]. Profound microcephaly is a usual finding and patients will usually die before they reach the age of 7 years. Mutation in the palmitoyl-protein thioesterase (*PPT*) gene which encodes a fatty acid cleavage lysosomal protein is the cause of NCL1. R122W (c.364A>T) and R151X (c.451C>T) are the most common mutations of the *PTT* gene and accounted for approximately 20% of mutations [2]. NCL2, also called Jansky–Bielschowsky disease, in which almost the majority of affected patients’ manifestations begin at late infantile period of life, is caused by mutations in the *CLN2* gene which encodes tripeptidyl-peptidase 1 (TPP1) [24]. NCL3 is the juvenile form of this disease and often diagnosed with retinitis pigmentosa and late onset seizures at the second decade of life [25]. There are other various types of NCLs; some of these conform to an autosomal dominant pattern of inheritance (types of NCL4 which are caused by mutation in the *DNAJC5* gene). NCL10 is the most malignant form and the only type of NCL which affect newborns [26–28]. NCL7 is caused by mutations in the *MFSD8*/*CLN7* gene and is a LINCL type [27, 28].

## Case presentation

**Fig. 1:**
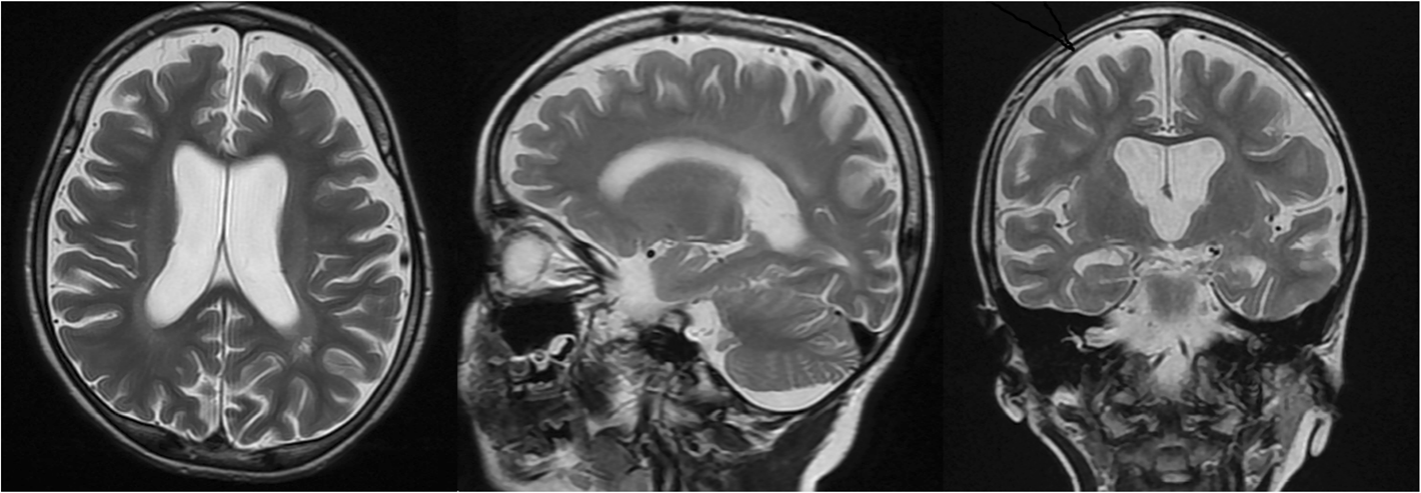
Brain magnetic resonance imaging of patient

**Fig. 2:**
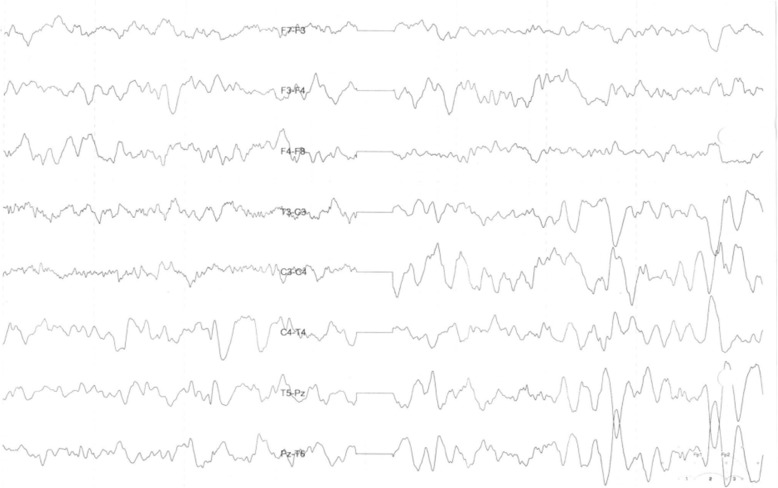
Brain electroencephalogram of patient

A 5-year-old Iranian boy with a neurodegenerative disorder was referred for genetic testing. His clinical symptoms were speech problems, lack of concentration, walking disability at age of 4 years leading to quadriplegia, spontaneous laughing and crying because of hidden seizure, clumsiness, psychomotor delay, and vision deterioration at age of 5 years which could be the consequence of macular dystrophy. Brain magnetic resonance imaging (MRI) and electroencephalogram (EEG) showed bilateral white matter signal change with preservation of white matter (Fig. 1 and Fig. 2). There are no available histopathological studies or skin biopsy for this patient.

The organic acids in his urine were determined using gas chromatography–mass spectrometry (GC-MS) of the trimethylsilyl ethers and esters of the ethyl acetate extract from acidified urine after preparation of the ethoxime derivatives. The organic acid in the urine of this patient showed a normal pattern with no evidence for metabolic disorders. There is no evidence for tyrosinemia, glutaric aciduria, methylmalonic aciduria, Canavan disease, propionic aciduria, isovaleric aciduria, and other organic aciduria known in Iran. Neonatal screening, clinical chemistry, and metabolism assays showed a normal pattern and these analyses were unremarkable (Table 2 and Table 3).

**Table 2:** Neonatal screening

Assay	Result	Reference range
Neonatal screening		
Thyroid-stimulating hormone (TSH)	< 10 mU/l	< 10 mU/l
17-OH progesterone	< 5 nmol/l	< 5 nmol/l
Galactose (GAL; GAL-1-P)	< 18 mg/dL	< 18 mg/dL
Succinylacetone	< 5 μmol/l	< 5 μmol/l
GAL-1-P uridyltransferase	> 20% activity	> 20% activity
Biotinidase	> 30% activity	> 30% activity
Disorder of amino acid metabolism		
Amino acids (including phenyl.) TMS		
Disorders of beta oxidation of fatty acids		
MCADD	Unremarkable	
VLCADD	Unremarkable	
LCHADD	Unremarkable	
Disorders of carnitine metabolism		
Acylcarnitines. TMS	Unremarkable	
Disorders of organic acids		
Isovalerylcarnitine	Unremarkable	
Glutaric acid	Unremarkable	
Defects of urea cycle		
Citrulline	Unremarkable	
Argininosuccinate	Unremarkable	

*act*, *Gal-1-P* galactose-1-phosphate, *LCHADD* long-chain 3-hydroxyacyl-CoA dehydrogenase deficiency, *MCADD* medium-chain acyl-CoA dehydrogenase deficiency, *phenyl* phenylalanine, *TMS* tandem mass spectrometry, *VLCADD* very long-chain acyl-CoA dehydrogenase deficiency

**Table 3:** Clinical chemistry and metabolism assays

Assay	Result	Reference range
Creatinine	0.89 g/l	0.12–1.12 g/l
Docosanoic acid (C22)	45.8 nmol/ml	15–113 nmol/ml
Tetracosanoic acid (C24)	41.6 nmol/ml	12–94 nmol/ml
Hexacosanoic acid (C26)	0.4 nmol/ml	0.2–1.6 nmol/ml
C24/C22	0.91	0.55–1.05
C26/C22	0.009	0.005–0.029
Phytanic acid	3.2 nmol/ml	0.3–31 nmol/ml
Aryl sulfatase A	0.808 mu/mg	0.375–1.815
Aryl sulfatase B	1.654 mu/mg	0.722–3.749

**Fig. 3:**
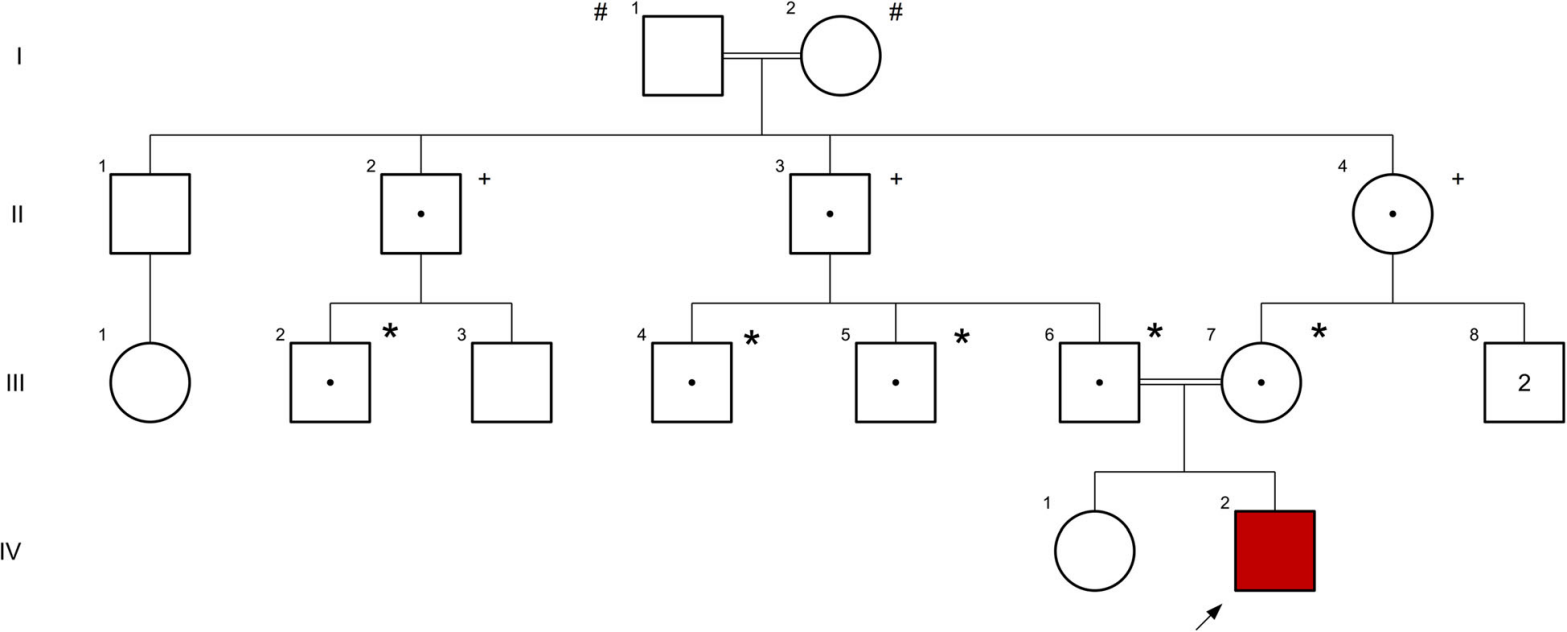
Pedigree of family affected by neuronal ceroid lipofuscinosis. *# de novo* or inherited deletion, *+ heterozygous* inferred from pedigree, * heterozygous confirmed by deletion analysis or Sanger sequencing. The *arrow* shows the proband in the family

Our patient’s parents are first cousins and had experienced three gestations, the first one aborted spontaneously before 4 weeks of pregnancy. The second one is the male reported here and the third one is a 2-year-old girl who does not manifest any signs and symptoms yet and seems to be normal. The pedigree is shown in Fig. 3.

Deoxyribonucleic acid (DNA) was extracted from peripheral blood of our patient and his healthy parents. Whole exome sequencing (WES) was performed on all three samples as following. Approximately 37 mega base pairs (Mb; 214,405 exons) of the Consensus Coding Sequences (CCS) were enriched from fragmented genomic DNA by > 340,000 probes designed against the human genome (Nextera Rapid Capture Exome, Illumina) and the generated library sequenced on an Illumina HiSeq 4000 platform (Illumina) to an average coverage depth of more than 100 × (Table 4). An end-to-end bioinformatics pipeline including base calling, primary filtering of low quality reads and probable artifacts, and annotation of variants was applied. All disease-causing variants reported in HGMD® or ClinVar (class 1) as well as all variants with minor allele frequency (MAF) of less than 1% in Exome Aggregation Consortium (ExAC) database were considered. The evaluation focused on exons and intron boundaries +/− 20. All relevant inheritance patterns were considered and clinical information was used to evaluate eventually identified variants. Relevant variants identified by WES were validated by Sanger sequencing in forward and reverse direction. By applying different filtering steps mentioned in the method part we ended up with only two novel variants in *MFSD8* and *AFF2* genes (Table 5).

**Table 4:** Analysis statistics, average coverage and % target base pairs covered

		% target bp covered					
	Average coverage (X)	0X	≥ 1X	≥ 5X	≥ 10X	≥ 20X	≥ 50X
Index	111.69	0.17	99.83	99.34	98.57	96.51	85.37
Mother	101.42	0.33	99.67	98.95	97.98	95.36	82.09
Father	106.15	0.19	99.81	99.25	98.32	95.86	83.15

*bp* base pairs

**Table 5:** Results of trio whole exome sequencing and significant findings

Gene (transcript)	Nucleotide (protein)	Zygosity			Described by	*In silico* parameters*	MAF**	Variant classification***	Disorder (OMIM# inheritance)
		Index	Mother	Father					
*AFF2* (NM_002025.3)	c.259A>G (p.Asn87Asp)	Hem.	Het.	–	Not described	2/4 Damaging	Not detected	Significance uncertain (class 3)	*AFF2*-related X-linked mental retardation (309,548, XLR)
*MFSD8* (NM_152778.2)	c.325_339del (p.Val109_Ile113del)	Hom.	Het.	Het.	Not described	In-frame small deletion	Not detected	Significance uncertain (class 3)	Neuronal ceroid lipofuscinosis type 7(610,951, AR)

* number of *in silico* prediction programs that predict pathogenicity/all applicable programs (SIFT, PolyPhen-2, AlignGVD, MutationTaster), ** highest minor allele frequency (MAF) of representative population – Exome Aggregation Consortium (ExAC) database, Exome Sequencing Project (ESP), or 1000 Genomes Project (1000G), *** based on ACMG recommendations, *ACMG* American College of Medical Genetics and Genomics, *AR* autosomal recessive, *hem* hemizygous, *het* heterozygous, *hom* homozygous, *MAF* minor allele frequency, *OMIM* Online Mendelian Inheritance in Man, *XLR* X-Linked Recessive

**Fig. 4:**
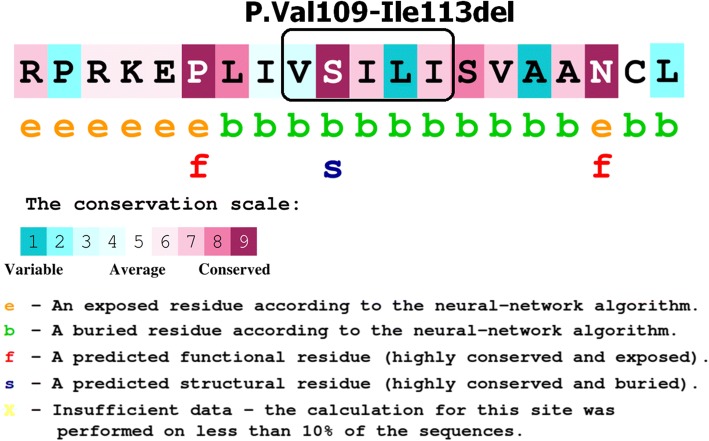
The conservation scale adapted from the ConSurf Service. http://consurf.tau.ac.il/2016/. Mutated domain highlighted in *box*

The c.325_339del (p.Val109_Ile113del) variant in the *MFSD8* gene was a previously unreported variant and found to be homozygous in our patient whereas his parents were heterozygote carriers (Table 5). Segregation analysis of this variant was done in the affected index, his parents, and relatives. It is a deletion of 15 base pairs (bp), which causes the loss of five residues. The evolutionary conservation of amino acid residues in the region of deletion estimated by ConSurf tool [29] is shown in Fig. 4.

The c.259A>G (p.Asn87Asp) variant found in *AFF2* was a hemizygous change in our patient; his mother was also heterozygous whereas his father was negative for this variant. This variant had not been reported in the public databases at the time of this publication (Table 5).

## Discussion

The identified variant is a 15 bp in-frame deletion in *MFSD8* gene. Pathogenic variants in the *MFSD8* gene have been shown to be associated with AR NCL type 7. Visual loss and seizures may be the initial signs and symptoms. Epilepsy, ataxia, and myoclonus may be the initial features [28] in children with age of onset after 4 years (Tables 6 and 7). Segregation analysis in this family found 15 bp in-frame deletion in *MFSD8* gene which is compatible with disease (Fig. 3).

**Table 6:** *AFF2* and *MFSD8* genes information and aliases

Gene	Ensembl/OMIM ID/Gene Ontology/	Aliases	Location/Exon count	Function
*MFSD8* (NM_152778.2)	ENSG00000164073/ 611,124/ GO:0007040 GO:0055085	Major facilitator superfamily domain-containing 8, *CLN7*	4q28.2/ 13 Exons	Transmembrane ubiquitous protein consists of major facilitator superfamily and transporter domains
*AFF2* (NM_002025.3)	ENSG00000155966/ 300,806/ GO:0002151	*AF4*/*FMR2* family member 2, fragile XE mental retardation syndrome protein, fragile X mental retardation 2 protein, protein FMR-2, *FMR2P*, *FMR2*, *OX19*, fragile X mental retardation 2, *AF4*/*FMR2* family member 2	Xq28/ 22 Exons	G-quadruplex RNA binding, RNA splicing

*OMIM* Online Mendelian Inheritance in Man

RNA Ribonucleic acid

**Table 7:** Common reported mutations of *AFF2* and *MFSD8* genes, related signs and symptoms and disorders

Gene	Common reported mutations	Signs and symptoms	Related disorders
*MFSD8* (NM_152778.2)	Missense and nonsense mutation, splice site mutation, frameshift, deletion	Seizures or motor impairment, mental regression, myoclonus seizure, speech impairment, loss of motor function and quadriplegia, early onset loss of vision, sleep disorders, loss of cognitive function	Ceroid lipofuscinosis, neuronal, 7
*AFF2* (NM_002025.3)	Triple expansion of GCC to > 200 in comparison with 6–25 normal copy number of GCC	Mild-to-moderate mental retardation, learning disabilities, communication problems, attention loss, hyperactivity, autistic behaviors, metabolic and homeostatic abnormalities	Fragile XE syndrome, X-linked mental retardation associated with fragile site FRAXE, FRAXE intellectual disability

*FRAXE* fragile XE syndrome

**Fig. 5:**
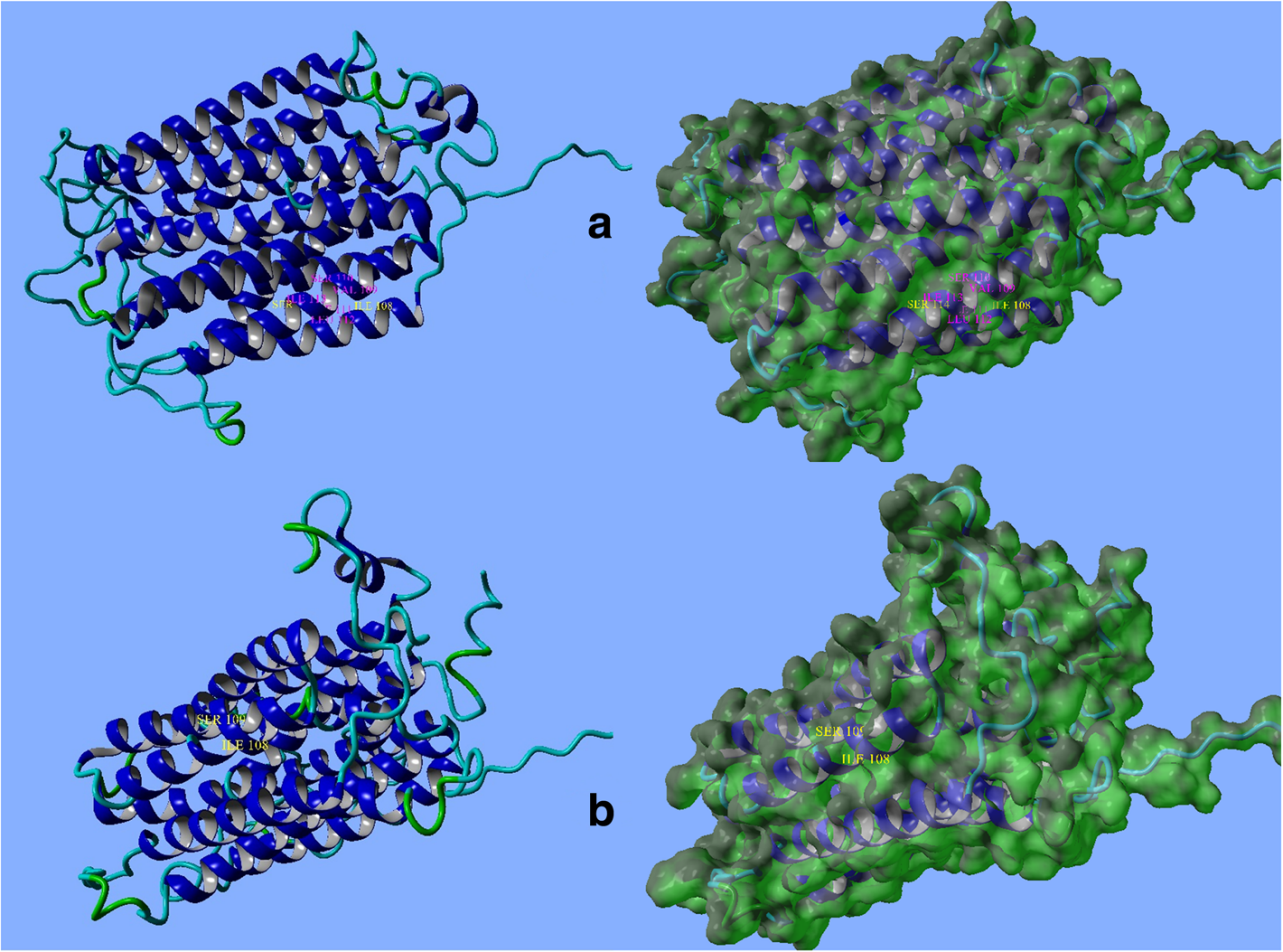
Front view and surface of MFSD8 protein. **a** Normal; **b** mutant. Structural topology has been changed in mutant form. Adapted from Yet Another Scientific Artificial Reality Application (YASARA)

*MFSD8* is located on chromosome 4q28.1-q28.2; it has 55,180 bp and 13 exons, encoding a 4562 bp transcript and a protein with 518 amino acids. Major facilitator superfamily domain-containing protein 8 which encoded by *MFSD8* is a transmembrane ubiquitous protein that consists of a major facilitator superfamily (MFS) and transporter domains [27]. This protein locates into lysosomal membrane and acts as a multi-pass membrane protein using chemiosmotic ion gradients [30]. Figure 5 shows topological structure of MFSD8 wild type and mutant protein. The deletion found in this patient affects the exon 5 of this gene which is the region encoding transmembrane domain. According to IRANOME database (http://www.iranome.ir/), the mean allele frequency of this variant is unknown.

Based on the NCL mutation database held at the University College London, at the time of writing this manuscript, there were 38 reported mutations in *MFSD8* gene in different populations [9]. The most common mutations are missense and nonsense mutations; however, other types of mutations such as splice site, single bp deletion, frameshift, and one case of 17 bp deletion are also reported [28].

Rare pathogenic variants in the AFF2 gene have been identified in patients with autism and attention deficit hyperactivity disorders as well as with X-linked intellectual disability and developmental and speech delay [31, 32]. In addition, this gene has been associated with syndromic autism, where a subpopulation of individuals with a given syndrome develops autism. A rare pathogenic variant in the *AFF2* gene has been identified in patients with fragile X syndrome [32]. Mondal *et al.* suggested that rare variants in *AFF2* may be the cause for previously unrecognized autism spectrum disorder (ASD) susceptibility locus and may help to explain some of the male excess of ASD [33]. The other related phenotypes to *AFF2* gene are abnormality of metabolism/homeostasis [34], aggressive behavior [35], epicanthic fold, and delayed speech and development of language [36].

*AFF2* is a protein coding gene and member of the *AF4*\*FMR2* gene family which is located on chromosome Xq28. This ribonucleic acid (RNA)-binding protein is implicated in the splicing process. The trinucleotide repeat expansion of CCG at 5′ untranslated region (UTR) of *AFF2* causes fragile XE syndrome (FRAXE) [37]. More details of this gene are included in Tables 6 and 7. Although the hemizygous variant found in the affected male of this family segregates in the tested samples, based on the clinical symptoms of the affected male, this variant cannot explain the hallmark and pathognomonic characteristics observed in the affected male. So, based on the clinical findings, correlation of the *AFF2* variant with the disease in this family is very unlikely. Tables 6 and 7 summarize the expected and observed phenotypic symptoms.

## Conclusions

**Fig. 6:**
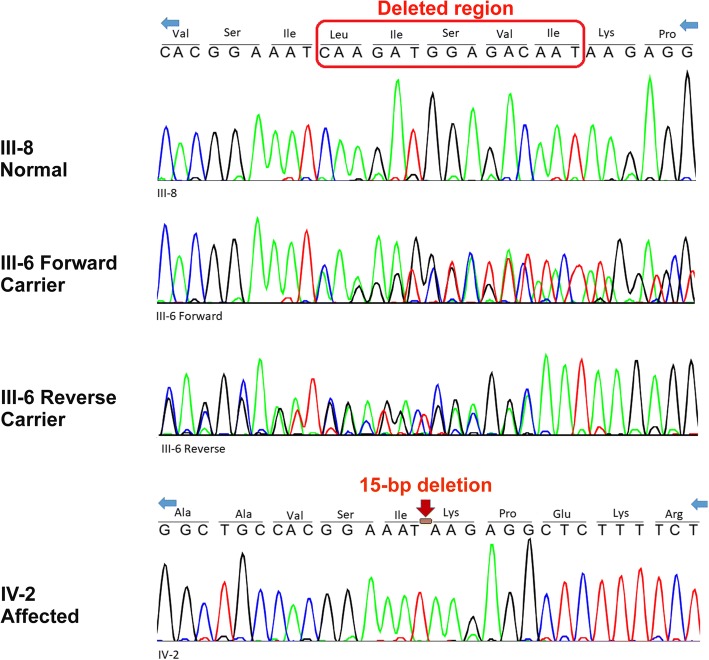
Deoxyribonucleic acid sequence of mutated region. Normal homozygous uncle and heterozygous parents and homozygous patient for in-frame 15 base pairs deletion. *bp* base pairs

Sequencing analysis in this family has shown that the index is homozygous for 15 bp in-frame deletion, his uncle has normal homozygous, and his parents are heterozygous (Fig. 6).

This pattern of mutation inheritance and the signs and symptoms observed in the affected male of this family are compatible with what is described in the literature for NCL type 7 and, therefore, suggest that the *MFSD8* deletion found in this study is most probably the cause of disease in this family.

## Abbreviations

AR: Autosomal recessive
ASD: Autism spectrum disorder
bp: Base pair
CCS: Consensus Coding Sequences
CLNs or NCLs: Neuronal ceroid lipofuscinoses
EEG: Electroencephalogram
FRAXE: Fragile XE syndrome
GC-MS: Gas chromatography–mass spectrometry
INCL: Infantile neuronal ceroid lipofuscinosis
JNCL: Juvenile neuronal ceroid lipofuscinosis
LINCL: Late infantile neuronal ceroid lipofuscinosis
MAF: Minor allele frequency
Mb: Mega base pairs
MFS: Major facilitator superfamily
NCLs or CLNs: Neuronal ceroid lipofuscinoses
*PPT*: Palmitoyl-protein thioesterase
TPP1: Tripeptidyl-peptidase 1
UTR: Untranslated region
WES: Whole exome sequencing

## References

[1] Geraets RD, Koh SY, Hastings ML, Kielian T, Pearce DA, Weimer JM. Moving towards effective therapeutic strategies for Neuronal Ceroid Lipofuscinosis. Orphanet J. Rare Dis. 2016;11(1):40. 10.1186/s13023-016-0414-2 PMC4833901 27083890

[2] Bennett MJ, Rakheja D. The neuronal ceroid-lipofuscinoses. Dev. Disabil. Res. Rev. 2013;17(3):254–9. 10.1002/ddrr.1118 23798013

[3] Jadav RH, Sinha S, Yasha TC, Aravinda H, Gayathri N, Rao S, Bindu PS, Satishchandra P. Clinical, electrophysiological, imaging, and ultrastructural description in 68 patients with neuronal ceroid lipofuscinoses and its subtypes. Pediatr Neurol. 2014;50(1):85–95. 10.1016/j.pediatrneurol.2013.08.008 24120650

[4] Albert D, De Los RE, Vidaurre J. A case of neuronal Ceroid Lipofuscinosis masquerading as Panayiotopoulos syndrome. J Pediatr Epilepsy. 2016;5(01):047–52.

[5] Stogmann E, El Tawil S, Wagenstaller J, Gaber A, Edris S, Abdelhady A, Assem-Hilger E, Leutmezer F, Bonelli S, Baumgartner C, Zimprich F. A novel mutation in the *MFSD8* gene in late infantile neuronal ceroid lipofuscinosis. Neurogenetics. 2009;10(1):73–7. 10.1007/s10048-008-0153-1 18850119

[6] Mink JW, Augustine EF, Adams HR, Marshall FJ, Kwon JM. Classification and natural history of the neuronal ceroid lipofuscinoses. J Child Neurol. 2013;9:0883073813494268. 10.1177/0883073813494268 PMC3979348 23838030

[7] Haltia M, Goebel HH. The neuronal ceroid-lipofuscinoses: a historical introduction. Biochim Biophys Acta. 2013;1832(11):1795–800. 10.1016/j.bbadis.2012.08.012 22959893

[8] Goebel HH, Mole SE, Lake BD. The neuronal ceroid lipofuscinoses (Batten disease). Amsterdam: IOS Press; 1999.

[9] NCL mutation database. Available at: https://www.ucl.ac.uk/ncl/SummaryTableMay2015.htm.

[10] Warrier V, Vieira M, Mole SE. Genetic basis and phenotypic correlations of the neuronal ceroid lipofuscinoses. Biochim Biophys Acta. 2013;1832(11):1827–30. 10.1016/j.bbadis.2013.03.017 23542453

[11] Sleat DE, Gin RM, Sohar I, Wisniewski K, Sklower-Brooks S, Pullarkat RK, Palmer DN, Lerner TJ, Boustany RM, Uldall P, Siakotos AN. Mutational analysis of the defective protease in classic late-infantile neuronal ceroid lipofuscinosis, a neurodegenerative lysosomal storage disorder. Am J Hum Genet. 1999;64(6):1511–23. 10.1086/302427 PMC1377895 10330339

[12] Mole SE, Williams RE, Goebel HH. Correlations between genotype, ultrastructural morphology and clinical phenotype in the neuronal ceroid lipofuscinoses. Neurogenetics. 2005;6(3):107–26. 10.1007/s10048-005-0218-3 15965709

[13] Jalanko A, Braulke T. Neuronal ceroid lipofuscinoses. Biochim Biophys Acta. 2009;1793(4):697–709. 10.1016/j.bbamcr.2008.11.004 19084560

[14] Sleat DE, Donnelly RJ, Lackland H, Liu CG, Sohar I, Pullarkat RK, Lobel P. Association of mutations in a lysosomal protein with classical late-infantile neuronal ceroid lipofuscinosis. Science. 1997;277(5333):1802–5. 10.1126/science.277.5333.1802 9295267

[15] Nita DA, Mole SE, Minassian BA. Neuronal ceroid lipofuscinoses. Epileptic Disord. 2016;18(s2):73–88. 10.1684/epd.2016.0844 27629553

[16] Götzl JK, Mori K, Damme M, Fellerer K, Tahirovic S, Kleinberger G, Janssens J, van der Zee J, Lang CM, Kremmer E, Martin JJ. Common pathobiochemical hallmarks of progranulin-associated frontotemporal lobar degeneration and neuronal ceroid lipofuscinosis. Acta Neuropathol. 2014;127(6):845–60. 10.1007/s00401-014-1262-6 24619111

[17] Karaa A, Simas AM, John S, Glykys J, Xin W, Cotman SL, Sims KB. Expanding the clinical spectrum of the lysosomal disorders with whole exome sequencing. Abstracts/Mol. Genet. Metab. 2015;114:S11–30.

[18] Patel J, Mercimek-Mahmutoglu S. Epileptic encephalopathy in childhood: a stepwise approach for identification of underlying genetic causes. Indian J. Pediatr. 2016;83(10):1164–74. 10.1007/s12098-015-1979-9 26821542

[19] García-Cazorla A, Wolf NI, Mochel F, Hoffmann GF. Neurological disease. In: Hoffman GF, Zschocke J, Nyhan WL, editors. Inherited metabolic diseases: A clinical approach. Berlin Heidelberg: Springer; 2017. p. 251–92.

[20] Langereis EJ, Wijburg FA. Lysosomal diseases and therapeutic options: an overview. In: Boelens JJ, Wynn R, editors. Stem cell therapy in Lysosomal storage diseases. New York: Springer; 2013. p. 1–20.

[21] Cooper JD, Williams RE. Neuronal Ceroid Lipofuscinoses. In: Mehta A, Winchester B, editors. Lysosomal Storage Disorders: A Practical Guide, vol. 4; 2012. p. 137–41.

[22] Järvelä I, Schleutker J, Haataja L, Santavuori P, Puhakka L, Manninen T, Palotie A, Sandkuijl LA, Renlund M, White R, Aula P. Infantile form of neuronal ceroid lipofuscinosis (CLN1) maps to the short arm of chromosome 1. Genomics. 1991;9(1):170–3. 10.1016/0888-7543(91)90235-7 1672288

[23] Grisolia M, Sestito S, Ceravolo F, Invernizzi F, Salpietro V, Polizzi A, Ruggieri M, Garavaglia B, Concolino D. The neuronal Ceroid Lipofuscinoses: a case-based overview. J Pediatr Biochemistry. 2016;6(01):060–5.

[24] Mahmood F, Fu S, Cooke J, Wilson SW, Cooper JD, Russell C. A zebrafish model of CLN2 disease is deficient in tripeptidyl peptidase 1 and displays progressive neurodegeneration accompanied by a reduction in proliferation. Brain. 2013;136:1488–507. 10.1093/brain/awt043 23587805

[25] Minye HM, Fabritius AL, Vesa J, Peltonen L. Data on characterizing the gene expression patterns of neuronal ceroid lipofuscinosis genes: *CLN1*, *CLN2*, *CLN3*, *CLN5* and their association to interneuron and neurotransmission markers: Parvalbumin and Somatostatin. Data in Brief. 2016;8:741–9. 10.1016/j.dib.2016.06.027 PMC4950137 27508227

[26] Mole SE, Goyal S, Williams RE. The neuronal Ceroid Lipofuscinoses. In: Panayiotopoulos CP, editor. Atlas of epilepsies. London: Springer; 2010. p. 1235–41.

[27] Kashyap SS, Johnson JR, McCue HV, Chen X, Edmonds MJ, Ayala M, Graham ME, Jenn RC, Barclay JW, Burgoyne RD, Morgan A. *Caenorhabditis elegans dnj-14*, the orthologue of the *DNAJC5* gene mutated in adult onset neuronal ceroid lipofuscinosis, provides a new platform for neuroprotective drug screening and identifies a SIR-2.1-independent action of resveratrol. Human Molecular Genetics. 2014;23:5916–27. 10.1093/hmg/ddu316 PMC4204773 24947438

[28] Craiu D, Dragostin O, Dica A, Hoffman-Zacharska D, Gos M, Bastian AE, Gherghiceanu M, Rolfs A, Nahavandi N, Craiu M, Iliescu C. Rett-like onset in late-infantile neuronal ceroid lipofuscinosis (CLN7) caused by compound heterozygous mutation in the *MFSD8* gene and review of the literature data on clinical onset signs. Eur J Paediatr Neurol. 2015;19(1):78–86. 10.1016/j.ejpn.2014.07.008 25439737

[29] Ashkenazy H, Erez E, Martz E, Pupko T, Ben-Tal N. ConSurf 2010: calculating evolutionary conservation in sequence and structure of proteins and nucleic acids. Nucleic Acids Res. 2010;38:W529–33. 10.1093/nar/gkq399 PMC2896094 20478830

[30] Aiello C, Terracciano A, Simonati A, Discepoli G, Cannelli N, Claps D, Crow YJ, Bianchi M, Kitzmuller C, Longo D, Tavoni A. Mutations in *MFSD8*/*CLN7* are a frequent cause of variant-late infantile neuronal ceroid lipofuscinosis. Hum Mutat. 2009;30(3):E530–40. 10.1002/humu.20975 19177532

[31] Abrams MT, Doheny KF, Mazzocco MM, Knight SJ, Baumgardner TL, Freund LS, Davies KE, Reiss AL. Cognitive, behavioral, and neuroanatomical assessment of two unrelated male children expressing FRAXE. Am J Med Genet. 1997;74(1):73–81. 10.1002/(sici)1096-8628(19970221)74:1<73::aid-ajmg16>3.0.co;2-o 9034011

[32] Bensaid M, Melko M, Bechara EG, Davidovic L, Berretta A, Catania MV, Gecz J, Lalli E, Bardoni B. FRAXE-associated mental retardation protein (FMR2) is an RNA-binding protein with high affinity for G-quartet RNA forming structure. Nucleic Acids Research. 2009;37:1269–79. 10.1093/nar/gkn1058 PMC2651778 19136466

[33] Mondal K, Ramachandran D, Patel VC, Hagen KR, Bose P, Cutler DJ, Zwick ME. Excess variants in *AFF2* detected by massively parallel sequencing of males with autism spectrum disorder. Hum Mol Genet. 2012;21(19):4356–64. 10.1093/hmg/dds267 PMC3441129 22773736

[34] Weinstein E, Cui X, Simmons P, Sigma-Aldrich Co. Genomic editing of genes involved in autism spectrum disorders. United States patent application US 12/842,678. 2010.

[35] Todorova A, Litvinenko I, Todorov T, Tincheva R, Avdjieva D, Tincheva S, Mitev V. A family with fragile X syndrome, Duchenne muscular dystrophy and ichthyosis transmitted by an asymptomatic carrier. Clin Genet. 2014;85(3):286–9. 10.1111/cge.12148 23574351

[36] Sahoo T, Theisen A, Marble M, Tervo R, Rosenfeld JA, Torchia BS, Shaffer LG. Microdeletion of Xq28 involving the *AFF2* (*FMR2*) gene in two unrelated males with developmental delay. Am J Med Genet A. 2011;155(12):3110–5. 10.1002/ajmg.a.34345 22065534

[37] Santos CB, Lima C, Pimentel MM. A new PCR assay useful for screening of FRAXE/FMR2 mental impairment among males. Hum Mutat. 2001;18(2):157–62. 10.1002/humu.1165 11462240

